# Enhancing Influenza Detection through Integrative Machine Learning and Nasopharyngeal Metabolomic Profiling: A Comprehensive Study

**DOI:** 10.3390/diagnostics14192214

**Published:** 2024-10-04

**Authors:** Md. Shaheenur Islam Sumon, Md Sakib Abrar Hossain, Haya Al-Sulaiti, Hadi M. Yassine, Muhammad E. H. Chowdhury

**Affiliations:** 1 Department of Electrical Engineering, Qatar University, Doha 2713, Qatar; sumon3455.ms@gmail.com (M.S.I.S.); mah690@uregina.ca (M.S.A.H.); 2 Department of Biomedical Sciences, College of Health Sciences, Qatar University, Doha 2713, Qatar; haya.alsulaiti@qu.edu.qa; 3 Biomedical Research Center, Qatar University, Doha 2713, Qatar

**Keywords:** influenza diagnosis, metabolomics, stacking machine learning, nasopharyngeal swabs, model explainability

## Abstract

**Background/Objectives:** Nasal and nasopharyngeal swabs are commonly used for detecting respiratory viruses, including influenza, which significantly alters host cell metabolites. This study aimed to develop a machine learning model to identify biomarkers that differentiate between influenza-positive and -negative cases using clinical metabolomics data. **Method:** A publicly available dataset of 236 nasopharyngeal samples screened via liquid chromatography–quadrupole time-of-flight (LC/Q-TOF) mass spectrometry was used. Among these, 118 samples tested positive for influenza (40 A H1N1, 39 A H3N2, 39 Influenza B), while 118 were negative controls. A stacking-based model was proposed using the top 20 selected features. Thirteen machine learning models were initially trained, and the top three were combined using predicted probabilities to form a stacking classifier. **Results**: The ExtraTrees stacking model outperformed other models, achieving 97.08% accuracy. External validation on a prospective cohort of 96 symptomatic individuals (48 positive and 48 negatives for influenza) showed 100% accuracy. SHAP values were used to enhance model explainability. Metabolites such as Pyroglutamic Acid (retention time: 0.81 min, *m*/*z*: 84.0447) and its in-source fragment ion (retention time: 0.81 min, *m*/*z*: 130.0507) showed minimal impact on influenza-positive cases. On the other hand, metabolites with a retention time of 10.34 min and *m*/*z* 106.0865, and a retention time of 8.65 min and *m*/*z* 211.1376, demonstrated significant positive contributions. **Conclusions**: This study highlights the effectiveness of integrating metabolomics data with machine learning for accurate influenza diagnosis. The stacking-based model, combined with SHAP analysis, provided robust performance and insights into key metabolites influencing predictions.

## 1. Introduction

Influenza (also known as “the flu”) is a respiratory infection of the sinuses, the lungs, and the throat [1]. According to the World Health Organization (WHO), influenza remains a worldwide phenomenon, with an annual incidence of ~5–10% among adults and 20–30% among children [2]. In contrast to influenza B viruses, which primarily affect humans, influenza A viruses (IAVs) undergo an ongoing complex life cycle originating from a vast reservoir of zoonotic material. Antigenic drift is a hallmark of IAVs, enabling them to evolve swiftly through mutations and evade the human immune system [3,4,5]. Because early detection is essential for effective antiviral therapy, most individuals with influenza-like infections are ultimately provided with supportive care only. Clinical detection of influenza infection is difficult because the initial symptoms are shared with those of other respiratory infections. Rapid antigen tests, which are frequently combined with polymerase chain reaction PCR-based tests during the “flu” season, can facilitate rapid differentiation between influenza and other causes of acute respiratory infections. To ensure timely and effective intervention, the WHO recommends initiating anti-influenza treatments without waiting for laboratory test results [5,6]. Inadequate sensitivity has also impeded advances in rapid diagnostic tests and digital immunoassays for influenza; thus, novel strategies are necessary [7]. In the previous decade, molecular testing was credited with the revolutionary development in the field of infectious disease diagnosis and surveillance [8,9,10]. This generally applies to clinical microbiology and virology laboratories where PCR is widely utilized. Notwithstanding the rapidity and exceptional accuracy demonstrated by PCR techniques, significant limitations endure, including substantial expenditures, complex methodologies, the inability to differentiate active infection from latency or colonization, and a lack of sensitivity when implemented on direct patient specimens.

Metabolomics is a dynamic field of systems biology that focuses on the dynamic fluctuations in metabolite concentrations that occur within biological systems as a consequence of specific perturbations or stimuli [11]. Recent studies have evaluated the effectiveness of various strategies used to treat influenza-infected mice and human influenza patients, utilizing metabolomics and lipidomics approaches to better understand the underlying metabolic changes, which can aid in diagnostics and treatment optimization [12,13,14,15,16]. The researchers implemented spatial metabolomics to examine metabolic changes in the serum and lungs of rodents infected with influenza A virus, which revealed site-specific metabolic perturbations [17]. These discoveries underscore the significance of spatial methodologies in developing a better comprehension of the pathogenesis of respiratory diseases and potential therapeutic strategies. Using a mass spectrometry-based lipidomics approach, researchers have provided a systems-scale perspective on membrane lipid dynamics of infected human lung epithelial cells [18]. Prognostication of mechanical ventilation and ICU stay duration in critically ill coronavirus disease 2019 (COVID-19) patients can be achieved through the analysis of metabolic alterations induced by severe acute respiratory syndrome coronavirus 2 (SARS-CoV-2) [19]. It is possible to predict recovery patterns and effectively manage the disease with the early detection of metabolic alterations in these patients. Comparing the severity of COVID-19 in published data, the predictive models utilizing metabolites demonstrated superior performance to Acute Physiology and Chronic Health Evaluation II (APACHE II) scores. Additionally, metabolomics has also been used to diagnose influenza from nasopharyngeal swabs by detecting decreased levels of the biomarker pyroglutamate in the nasopharyngeal specimens of patients with influenza virus infection [20]. Finally, a pilot study of seasonal influenza vaccination showed that statistically significant metabolite features were found both for the vaccination effect and drug-vaccine interactions [21]. Metabolomics studies have great potential for diagnosing infectious diseases because of their capacity for direct, low-volume, cost-effective, and real-time evaluation of host responses [22,23]. They also enable differentiation between active infection and colonization. While metabolomics is primarily used for diagnostic purposes, it contributes indirectly to treatment by identifying biomarkers and metabolic pathways that could inform therapeutic targets and optimize personalized treatments.

Numerous deep learning strategies have recently been proposed as a means to formalize metabolomics as well.

A study was conducted by Chi et al. [24] to investigate acute ischemic stroke in 150 patients. To achieve this, the researchers analyzed blood samples collected over seven days using liquid chromatography and mass spectrometry. The study employed multivariate analysis to identify 63 metabolites that exhibited a complex association with functional outcomes. The efficacy of outcome prediction was significantly improved through the incorporation of machine learning techniques and the clinical characteristics of patients. A machine learning-based algorithm integrated with mass spectrometry was utilized in a cross-sectional study conducted by Jeany et al. [25] to differentiate COVID-19 in plasma samples with speed and precision, thereby addressing the ongoing challenges posed by the virus. Involving 815 patients from three Brazilian epicenters, the study demonstrated the method’s efficacy and potential practical utility in patient management and decision-making. Gerard et al. [26] utilized metabolomics techniques and machine learning algorithms to analyze the concentrations of metabolites in the serum and urine of patients with COVID-19.

The practical significance of influenza diagnostics, especially through metabolomics, lies in the early detection and precise understanding of metabolic changes that could guide targeted treatments beyond supportive care. During influenza infection, Vivian et al. [12] observed that cells increased glucose uptake and glycolysis, which fueled viral replication by producing ATP and essential intermediates. In the pentose phosphate pathway, viral proteins activated pathways such as mTORC1 and mTORC2, which in turn upregulated enzymes like G6PD, thereby providing energy for viral proliferation. Additionally, technological innovations in diagnostic methods, including rapid influenza diagnostic tests (RIDTs) [27], can enhance clinical management during epidemics by allowing healthcare professionals to distinguish between viral and bacterial infections. This, in turn, reduces the unnecessary use of antibiotics and encourages the development of more precise treatments. This also aids in the allocation of resources during virus seasons, thereby reducing hospital congestion by preventing unnecessary admissions.

Recently Hogan et al. [28] used liquid chromatography–quadrupole time-of-flight (LC/Q-TOF) and ML to diagnose influenza using nasopharyngeal swab samples. A set of 236 samples were analyzed in the ML model development phase. This was extended via a clinically applicable LC/MS analysis performed on a prospective cohort of samples from 96 symptomatic individuals. This metabolomics approach showed promise for diagnostic applications in infectious diseases, such as respiratory viruses, with future potential for point-of-care testing. In a more recent study that featured metabolomics and ML, Gerard et al. [26] analyzed the results of a GC/MS study of serum and urine metabolites in 126 patients diagnosed with COVID-19. They compared the results to samples from healthy individuals and patients diagnosed with bacterial infection. Diagnostic metabolites, including maltose and succinic acid, were identified in the serum samples, and the detection of Lauric acid correlated with the severity of infection. Analysis of urine samples revealed no valuable distinctions. This study employs targeted metabolomics and ML to explore potential diagnostic and prognostic biomarkers for COVID-19.

In this study, we applied supervised ML to a set of nasopharyngeal metabolomics samples collected by Hogan et al. [28]. The methods used in this study include collection of the nasopharyngeal samples, their subsequent analysis via LC/Q-TOF mass spectrometry (MS), alignment, peak selection, and normalization of the LC-MS dataset. The use of supervised ML permits the identification of metabolites with critical mass-to-charge (*m*/*z*) values and retention times, which in turn facilitates the accurate detection of influenza-related patterns. This study encompasses several pivotal aspects, including the following:

- This study utilized a nasopharyngeal metabolomics dataset that was publicly available, as reported by Hogan et al. [28], ensuring transparency and accessibility in data sourcing.
- The top 20 features were selected using Shapley Additive Explanations (SHAP) values.
- A stacking-based meta-classifier approach was proposed with 5-fold cross-validation. Initially, thirteen machine learning models were trained, and the three best models were selected based on their probabilities. These three models were combined to train 13 meta-classifier models.
- For the primary dataset, the ExtraTrees stacking model achieved the best results with 97.08% accuracy. External validation yielded 100% accuracy.
- Model explainability was utilized to identify the biomarkers responsible for influenza prediction.

## 2. Methods and Materials

**Figure 1:**
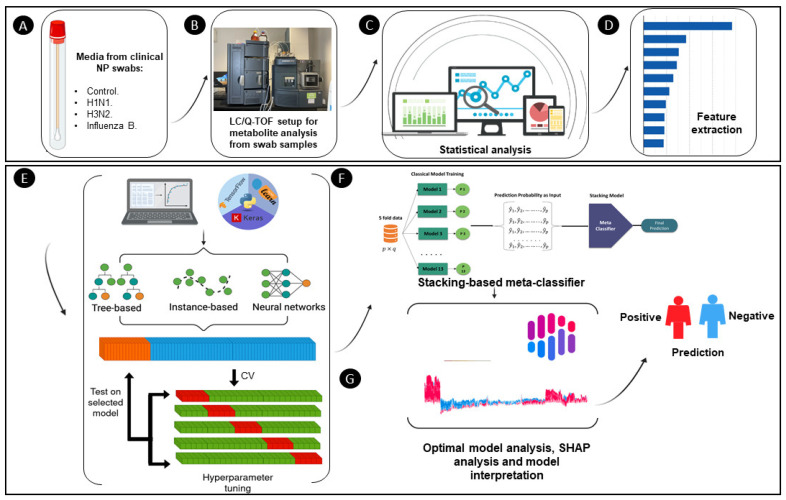
Schematic representation of the experimental framework. (**A**) Nasopharyngeal swabs collected for controls and different influenza strains (H1N1, H3N2, Influenza B). (**B**) LC/Q-TOF used to analyze the metabolic data from the swab samples. (**C**) Statistical analysis performed on the metabolomics data. (**D**) Feature extraction identifying key metabolites. (**E**) Model development using tree-based, instance-based, and neural networks, followed by hyperparameter tuning and cross-validation. (**F**) Stacking-based meta-classifier combining multiple models’ predictions. (**G**) Model interpretation using SHAP analysis and prediction outcomes as positive or negative for influenza.

The workflow process, as depicted in Figure 1, begins by leveraging a public dataset consisting of nasopharyngeal swab samples, which are analyzed using liquid chromatography–quadrupole time-of-flight mass spectrometry (LC/Q-TOF). This analysis facilitates the detection and quantification of metabolites from the swab samples, which is essential for understanding the underlying biological processes related to influenza infection. Following the acquisition of the metabolomics data, a variety of statistical analyses are performed to explore the dataset’s structure and patterns. Techniques such as t-distributed Stochastic Neighbor Embedding (t-SNE) and parallel coordinate plots are employed for dimensionality reduction and visualization of complex multivariate data, providing insights into the similarities and differences among sample groups (e.g., control, H1N1, H3N2, and Influenza B). Subsequently, feature selection is carried out to identify the most important metabolites influencing the classification outcomes. This step is accomplished using SHAP (Shapley Additive Explanations) values, a model-agnostic method that quantifies the contribution of each feature to the prediction. By ranking features according to their SHAP values, the most influential metabolites are identified, enhancing the interpretability of the model. The processed dataset is then subjected to 5-fold cross-validation, a technique used to ensure robust model evaluation by training the model on different subsets of the data while testing it on unseen samples. Thirteen machine learning models, spanning tree-based, instance-based, and neural network architectures, are initially trained on the dataset. Based on performance metrics such as accuracy, precision, recall, F1 score, and AUC, the top three models are selected for further optimization. The selected models are then integrated into a stacking-based meta-classifier. In this ensemble learning approach, the prediction probabilities generated by the three base models are used as input for a higher-level meta-model, which makes the final prediction. This stacking methodology aims to enhance the overall performance by leveraging the strengths of each base model. To ensure model interpretability and provide insights into the prediction process, SHAP values are once again computed for the final meta-model. This step allows for the identification of the key features (i.e., metabolites) that most significantly influence the model’s decision-making process, offering a transparent view of how predictions are generated and enabling the extraction of meaningful biological insights.

### 2.1. Description of the Dataset

The dataset that was employed in this study was acquired from Hogan et al. [28]. This group previously performed a comprehensive analysis of the nasopharyngeal swap samples using liquid chromatography–quadrupole time-of-flight mass spectrometry (LC/Q-TOF). The objective was to identify the metabolic signatures that led to a precise diagnosis of influenza. Two hundred and thirty-six samples were examined during the discovery phase. Of these, 118 were identified as positive (i.e., with signatures corresponding to Influenza A 2009 H1N1, Influenza H3N2, and Influenza B infection). An equivalent number of negative controls from age- and sex-matched patients with acute respiratory illness (H1N1, Flu B, and H3N2) were included in the analysis. An autonomous validation process was executed on a prospective cohort that included 96 individuals who were presented with relevant symptoms. This cohort included 48 positive samples (24 Influenza A 2009 H1N1, five Influenza H3N2, and 19 Influenza B) in addition to 48 negative samples.

**Figure 2:**
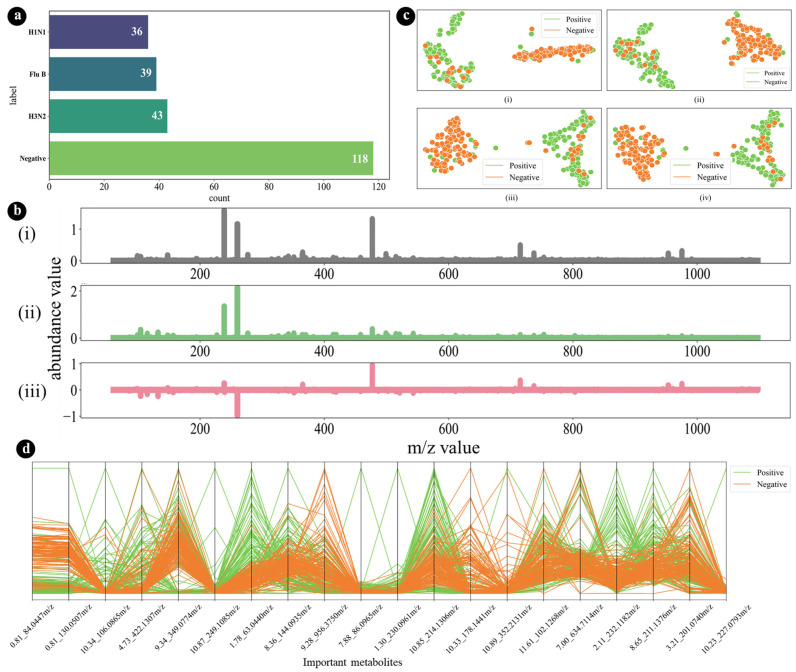
(**a**) The number of samples in each of the influenza-positive and influenza-negative groups. (**b**) Metabolomic profiles of (i) arbitrary samples from the original set of 118 that tested negative for influenza, (ii) arbitrary samples from this set that tested positive for influenza, and (iii) the difference between the selected samples, where the *x*-axis represents sequential mass-to-charge ratios, and the *y*-axis represents normalized relative abundance values. (**c**) Visualization of a ranked feature space based on the selected RandomForest feature of the ranking model for (orange dots represent positive samples and the other dots represent negative samples) (i) the top 5 ranked features (metabolites), (ii) the top 10 ranked features, (iii) the top 15 ranked features, and (iv) the top 20 ranked features using the manifold learning-based t-SNE technique. (**d**) Parallel coordinate plot of 20 selected feature spaces demonstrating class separability.

The LC/Q-TOF data collected from the nasopharyngeal samples included potential metabolomic signatures for patients with influenza infection and control (uninfected) patients. The sample counts for the group with influenza infection and the control group are illustrated in Figure 2a, together with examples of the metabolomic profiles of arbitrary influenza-infected-negative (control) and -positive samples (Figure 2b). It is difficult to distinguish between the metabolomic profiles based on simple visual inspection. However, when the differences between the two types of metabolomic profiles were plotted, we identified some fluctuation in the normalized abundance values.

It is vital to achieve a balance between the number of features and the number of samples to prevent overfitting or underfitting the model. For many “omics” problems, the feature number-to-sample size ratio is extremely unbalanced, which will inevitably lead to feature space complexity. Moreover, the primary goal of this analysis is the parsimonious identification of signature features. To accomplish this goal, we had to identify and isolate the most relevant and important features from the mass of data that was collected.

This necessity motivated us to apply several feature-ranking techniques to the original feature space, which included more than 3200 compounds (represented as mass-to-charge ratios with varying retention times). We used the SHAP method [29,30] to quantify the effects of the individual features identified in our models. This proved to be a vital component of our analysis. We focused on the top 20 ion features to identify those that were clinically significant ion features with global predictive power. The Shapley values for these characteristics were analyzed individually and collectively so that we gained a thorough comprehension of their combined effect. We reported the average absolute Shapley contributions as a proportion of the total feature contributions. The objective of this method was to identify distinguishing metabolites that could be classified with confidence for use in a prospective sample cohort.

By decreasing the dimension of the feature space, the feature selection techniques helped to increase the class separability. High dimensional manifold learning methods (e.g., Principal Component Analysis (PCA) [31] and t-distributed Stochastic Neighbor Embedding (t-SNE) [32]) were used to evaluate the quality of each of the reduced feature spaces derived from the various ranking techniques. A manifold learning-based high-dimensional visualization was used to select a single feature space for the classification networks. The t-SNE plots display various top-ranked features from the selected SHAP feature selection technique (Figure 2c). Figure 2(ci) demonstrates the efficacy of the t-SNE visualization, showcasing the discriminative capability of the selected 20 features to separate the two classes effectively. The parallel coordinate plot of the selected (or reduced) features space illustrates the high separability of the two investigated classes (i.e., influenza-positive and influenza-negative samples). The parallel coordinate plot shows that the sample contains a wide variety of compounds, with a wide range of *m*/*z* values. Several compounds have similar *m*/*z* values, but different intensities. This suggests that the sample contains a mixture of compounds with similar structures, which is also illustrated in Figure 2d.

We also employed an external validation dataset from Hogan et al. [28]. We prospectively selected nasopharyngeal and nasal swab specimens at a 1:1 ratio from those collected between 21 December 2019 and 18 February 2020. The samples were stored at −80 °C until tested. The complete set (total 96) included samples from 14 children and 80 adults, and three cases of documented viral coinfection (seasonal coronavirus, respiratory syncytial virus (RSV), or cytomegalovirus CMV)). These samples underwent LC/MS-MS analysis at Stanford’s Biochemical Genetics Laboratory. Supplementary Table S1 details the fundamental demographic characteristics of every patient who took part in the biomarker discovery (LC/Q-TOF) phase of the intervention.

### 2.2. Dataset Preprocessing

The metabolic profile of each sample included in the dataset was defined by its mass-to-charge ratio (*m*/*z*), retention time, and relative abundance. Preparing these data for incorporation into our ML models required meticulous attention to data preprocessing. This entailed a series of suitable techniques aimed at optimizing the database’s structure to facilitate practical training.

#### 2.2.1. Dataset Cleaning

**Figure 3:**
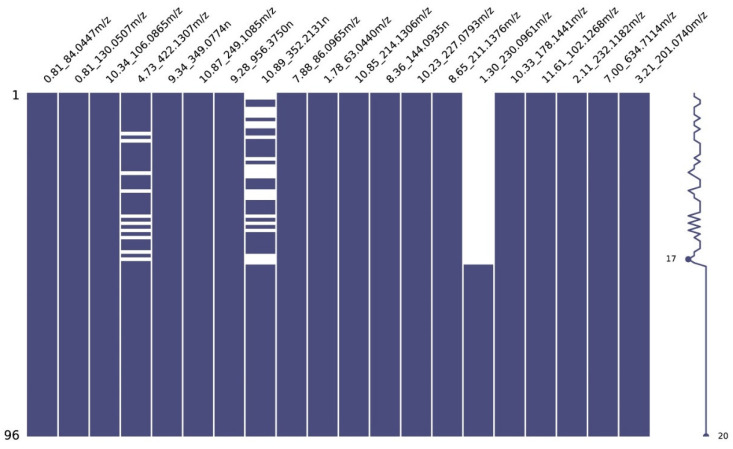
Missing data matrix with sparklines for different features in the external validation set.

A total of 248 samples underwent LC/Q-TOF analysis for metabolite discovery. Out of these, six were excluded because of technical errors, following the exclusion criteria outlined in the study by Hogan et al. [28]. Consequently, their corresponding six controls were also excluded. The final analysis encompassed 236 samples, comprising 118 positive influenza samples (40 Influenza A 2009 H1N1, 39 Influenza A H3N2, and 39 Influenza B) and an equal number of negative age- and sex-matched controls. Each sample was associated with 3366 metabolite features. Of these, 48 metabolite features were excluded from further analysis because they exhibited zero values in all clinical samples tested. Figure 3 represents a missing data matrix with sparklines for various features in the external validation set. Each column corresponds to a specific metabolite feature, identified by its mass-to-charge ratio (*m*/*z*), while each row represents an individual sample. Missing values are represented by white gaps in the matrix, indicating the absence of data for certain features. The sparklines on the right-hand side provide a visual summary of the distribution of missing data across samples for each feature, highlighting patterns and trends in data completeness. Feature 4.73_422.1307*m*/*z* has ten missing values, while 17 are missing from feature 1.30_230.0961*m*/*z*. Of note, the term “4.73” represents retention time, and “422.1307” signifies the *m*/*z* ratio. When generating metabolomics data for ML purposes, efforts to address these missing values become crucial for maintaining the integrity of instances of partially documented data. The systematic process of deducing missing values, known as data imputation, is effective for this purpose.

#### 2.2.2. Missing Data Imputation

For this investigation, the mean imputation method was selected as an approach to remedy the issue of missing data points [33,34]. This method substitutes any missing values with the average of the respective feature from all accessible samples. This is a straightforward and commonly employed method for addressing missing data, particularly in situations where the absence of data is random and not consistently skewed. The mean imputation method preserves sample size and guarantees dataset completeness for further analysis, mitigating the risk of bias that may arise from excluding samples with missing data. However, while effective, mean imputation can sometimes underestimate the variability in the data, which is why it is often used alongside other robust analysis methods to ensure the integrity of the results.

#### 2.2.3. Dataset Splitting

To train the machine learning model, we implemented a 5-fold cross-validation strategy on the 236-sample dataset. This method involved dividing the dataset into five distinct subsets. In each iteration, four subsets (approximately 80% of the data) were used for training, while the remaining subset (approximately 20% of the data) was used for testing. This process was repeated five times, ensuring that each subset was used as the test set exactly once, thereby maintaining a balanced distribution and enabling robust model evaluation.

#### 2.2.4. Normalization

Optimal training of an ML model relies on normalizing input data. This procedure improves the overall efficacy of training and ensures that each feature contributes proportionally. The entire dataset was scaled to a standard range via data normalization techniques such as StandardScaler. Results from several previous studies revealed that models trained on normalized data perform better than those built on unprocessed data. The dataset was normalized using StandardScaler, which facilitates robust model training and promotes system performance [35,36].

#### 2.2.5. Feature Selection

Feature selection is the process by which the features in a dataset that are most crucial for making accurate predictions in ML are identified. It is a crucial stage in numerous ML workflows, as appropriate feature selection can enhance model performance, decrease overfitting, and render models more comprehensible [37]. There are numerous approaches to ranking features, but SHAP values are among those used most frequently [38]. SHAP values can be used in a model-independent fashion to elucidate the predictions of any ML model. Using this method, the average effect of each feature on the prediction of the model is determined while taking into consideration all possible feature combinations. By prioritizing important features, ML models can achieve better accuracy, generalize more effectively, and offer insights into critical factors that influence predictions [39].

**Figure 4:**
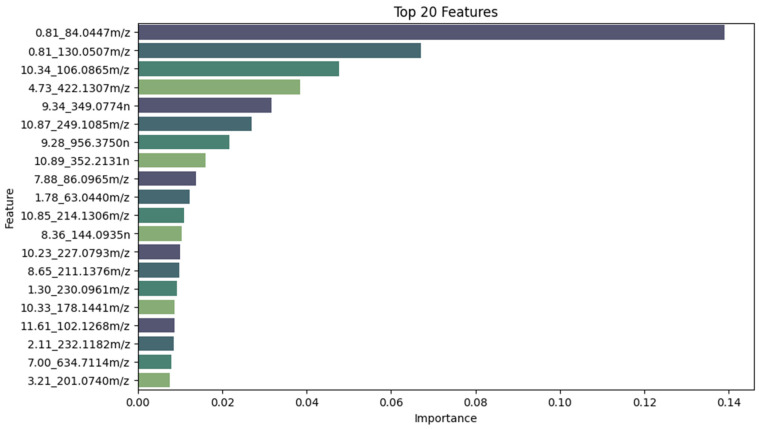
Top 20 features selected using Shapley Additive Explanation (SHAP) values.

In this study, an LGBM ML model was trained before feature selection with SHAP. SHAP values were calculated for each feature and observation in the dataset. The features were sorted by their average SHAP value in descending order, and the top 20 features were selected. These top 20 features are represented in Figure 4, which highlights the model’s key predictors and their impact on the metabolomics dataset. Each feature is identified by its retention time and mass-to-charge ratio, with the color in the visualization representing the association between feature values and positive influenza classification.

### 2.3. Development of Machine Learning Models

Classical ML models employ probabilistic and statistical techniques. These models have been in existence for many years and they have been utilized extensively and are well-understood [40]. As part of this research, we examined ML models from the Scikit Learn library.

#### Stacking-Based Meta-Classifier

We introduced a stacking approach [41] for the detection of influenza using metabolomics data in this study. To accomplish the ultimate objective of model training, our framework implemented a stacking algorithm. This design seamlessly integrates the attributes of the dataset with their respective ground truth outputs, combining several top-tier traditional machine learning models. Stacking, or stacked generalization, is an ensemble technique that combines predictions from multiple base models to enhance performance. It involves training diverse models, generating meta-features from their predictions, and using a meta-learner to combine these features for final predictions. This approach often leads to improved accuracy and reduced overfitting.

In this investigation part, thirteen classical machine learning algorithms were trained, which included Support Vector Machine (SVM) [42], Multilayer Perceptron (MLP) [43], Light Gradient Boosting Machine (LGBM), Logistic Regression (LR) [44], Gradient Boosting [45], Extreme Gradient Boosting (XGB) [46], Elastic Net, K-Nearest Neighbors (KNNs) [46], Categorical Boosting (CatBoost) [47], Adaptive Boosting (AdaBoost) [48], Linear Discriminant Analysis (LDA) [49], Extremely Randomized Trees (ExtraTree) [50], and Random Forest (RF) [51].

We employed five-fold cross-validation to generate a training set for the meta-level classifier. Each base-level classifier predicted a probability distribution over the possible class values. Utilizing input y and the predictions of the base-level classifier set M, a probability distribution was created as follows:

(1) $$P^{M}\left(y\right)=\left(P^{M}\left(c_{1}|y\right),P^{M}\left(c_{2}|y\right),\ldots \ldots .,P^{M}\left(c_{m}|y\right)\right)$$

where ($c_{1}$, $c_{2}$ … $c_{m}$) represents the set of potential class values and $P^{M}\left(c_{i}|y\right)$ presents the probability that example y belongs to class $c_{i}$, as calculated (and predicted) by classifier M [52,53]. In our investigation, the best models we identified were CatBoost, RandomForest, and AdaBoost. We utilized their probabilities to train stacking-based meta-models.

**Figure 5:**
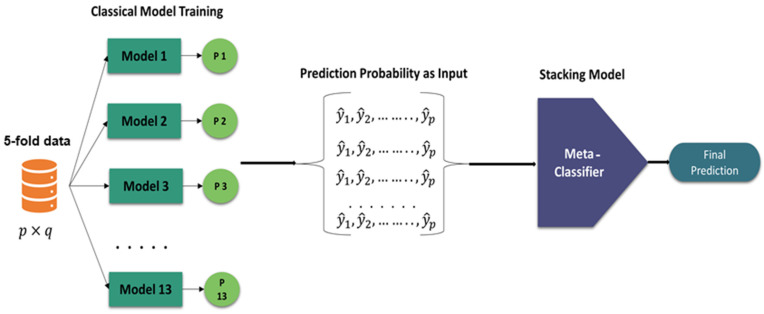
Framework of the proposed stacking machine learning algorithm.

Figure 5 illustrates the proposed stacking approach. The process begins with a classical model training phase using 5-fold cross-validation data, denoted as p×q. Thirteen different models (Model 1 through Model 13) are trained separately, and each model outputs a probability distribution ($P_{1}$ Through $P_{13}$) over the possible class values. These predicted probabilities serve as inputs to form a new dataset for the meta-classifier. The stacking model then utilizes these inputs to train the meta-classifier, which ultimately generates the final prediction. This approach leverages the strengths of multiple base-level classifiers to improve the overall prediction accuracy through a meta-level learning process.

### 2.4. Experimental Setup

The investigation utilized the Sklearn package in conjunction with Python 3.9.1. The specifications provided were utilized in the training of each model. All models were trained using Google ColabPro 279. ColabPro’s specifications included a 16 GB Tesla T4 280 GPU and 120 GB of high-speed RAM.

### 2.5. Performance Metrics

The performance evaluation was based on several key metrics, including Receiver Operating Characteristic (ROC) curves and the area under the curve (AUC). Additionally, precision, sensitivity, specificity, accuracy, and F1 score were employed as part of our comprehensive assessment. The evaluation metrics were denoted as shown in Equations (2) to (5):

(2) $$Accuracy_{class\_j}=\frac{TP_{class\_j}+TN_{class\_}j}{TP_{class\_j}+TN_{class\_j}+FP_{class\_j}+FN_{class\_j}}a$$

(3) $$Precision_{class\_j}=\frac{TP_{class\_j}}{TP_{class\_j}+FP_{class\_j}}$$

(4) $$Recall/Sensitivity_{class_{j}}=\frac{TP_{class_{j}}}{TP_{class_{j}}+FN_{class_{j}}}$$

(5) $$F1\_score_{class_{j}}=2\frac{Precision_{class_{j}}\times Sensitivity_{class_{j}}}{Precision_{class_{j}}+Sensitivity_{class_{j}}}$$

## 3. Results

In this section, we describe the results of two datasets based on the top 20 features identified using SHAP, as outlined in Section 2.2.5. Initially, the main internal dataset results were analyzed, followed by the validation of the model using an external dataset for further verification.

### 3.1. Internal Validation

Thirteen ML models were trained using a publicly available nasopharyngeal metabolomics dataset to predict influenza. From the classical models, the top three were selected based on their performance metrics and then used predicted probabilities to create a stacking-based classifier. We used the same classical models to train on the stacking algorithm, where all of them acted as the meta-classifier. The metabolomics dataset included 236 samples, with 118 testing positive for influenza. These positive samples were further categorized into 40 patients with Influenza A (2009 H1N1) infection, 39 with Influenza A (H3N2), and 39 with Influenza B. The remaining 118 samples were influenza-negative. We employed a five-fold cross-validation strategy, splitting the data into 80% training and 20% testing sets.

The results in Table 1 underscore the effectiveness of the stacking-based model compared with individual ML models. The stacking model consistently outperformed the individual models across all evaluated metrics, including accuracy, precision, recall, specificity, F1 score, and AUC. For instance, CatBoost’s accuracy improved from 94.58% to 95.83%, with its AUC increasing from 98.57% to 98.78%. Similarly, RandomForest’s accuracy rose from 94.16% to 96.25%, with an AUC improvement from 97.74% to 98.21%. Models such as ExtraTrees and SVM also exhibited significant enhancements, with ExtraTrees’ accuracy jumping from 93.75% to 97.08% and SVM’s from 92.50% to 95.83%. Overall, the stacking model improved accuracy by at least 2% compared with the initial models. The best stacking meta-model was ExtraTrees, which achieved an accuracy of 97.08%, precision of 97.11%, recall of 97.08%, specificity of 97.18%, F1 score of 97.08%, and an AUC of 98.12.

The ROC curves for the best stacking model across each fold are illustrated in Supplementary Figure S1. Notably, for folds 4 and 5, we achieved an AUC of 100%. The corresponding confusion matrices are presented in Supplementary Figure S2.

### 3.2. External Validation

External validation was conducted using the dataset collected by Hogan et al. [28]. To achieve this goal, nasopharyngeal and nasal swab specimens were prospectively selected at a 1:1 ratio between 21 December 2019 and 18 February 2020. These samples were preserved at −80 °C until analysis. The 96-sample set underwent LC/MS-MS analysis at Stanford’s Biochemical Genetics Laboratory. The 96 samples included 14 from children, 80 from adults, and 3 with documented viral coinfection (seasonal coronavirus, RSV, or CMV). We conducted external validation of the internal dataset using the top three performing models identified from Section 3.1, which included the following: CatBoost, RandomForest, and AdaBoost. Initially, these models were employed to generate probabilities on the external dataset. Subsequently, we employed a stacking ensemble approach with these models as base learners. The ExtraTrees stacking model demonstrated superior performance relative to the other models in Section 3.1. When evaluated on an external dataset, it achieved perfect scores of 100% across the accuracy, precision, recall, specificity, F1 score, and AUC (area under the curve) metrics.

### 3.3. Model Explainability

Interpreting complex models, especially ensemble or deep learning models, is crucial for understanding their outcomes and ensuring their applicability in real-world scenarios. Despite their high accuracy, these models present challenges in interpretation. To bridge this gap, various algorithms have been developed to aid in interpreting model outcomes. However, understanding the nuances and determining when to prioritize one approach over another remains a challenge. The tension between accuracy and interpretability underscores the need for comprehensive methods that balance both aspects effectively. Utilizing Shapley Additive Explanations (SHAP) [37] transcends the conventional understanding of model training and prediction generation. SHAP combines game theory with machine learning to offer precise insights into prediction mechanisms, thereby enabling a comprehensive understanding of outcomes.

The use of SHAP values allowed us to quantify the influence of individual features on the model’s predictions, thereby providing insights into the importance and contribution of each feature towards the classification outcomes. This approach ensured transparency and enhanced the interpretability of the model’s decision-making process.

**Figure 6:**
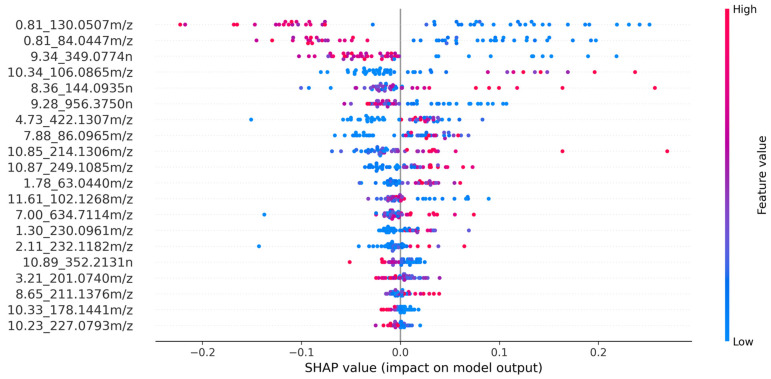
SHAP summary plot for the stacking model (ExtraTrees) highlighting feature impacts on the output.

We employed SHAP (Shapley Additive Explanations) to evaluate the impact of individual features on the model output for the stacking meta-model, ExtraTrees, as shown in Figure 6. These visual representations offer insights into the contribution of individual features towards model predictions, aiding in the interpretation of model outcomes. In the plot, each point represents a SHAP value for a specific feature in a particular instance. The *x*-axis indicates the SHAP value, which reflects the impact of the feature on the model’s prediction. Points to the right of the centerline (positive SHAP values) increase the probability of the positive class (influenza presence), whereas points to the left (negative SHAP values) decrease this probability. The color gradient from blue to red represents the feature value, with blue indicating low values and red indicating high values.

Figure 6 shows which features have the most impact on whether someone has influenza or not. The 0.81_84.0447*m*/*z* and 0.81_130.0507*m*/*z* features are more associated with not having influenza. Specifically, the 0.81_130.0507*m*/*z* feature, linked to Pyroglutamic Acid [28], decreases significantly in people with influenza, making it a potential marker for identifying those without the virus. Feature 0.81_84.0447*m*/*z* (In-Source Fragment Ion of Pyroglutamic Acid) [28] exhibits lesser contributions in the positive direction, suggesting a significant decrease in influenza-positive patients. In contrast, the 8.65_211.1376*m*/*z* feature has a stronger effect on people with influenza. Another important feature is 10.34_106.0865*m*/*z*, identified as 2-Amino-2-methyl-1,3-propanediol through the METLIN database [54], which notably detects influenza-positive cases.

**Figure 7:**
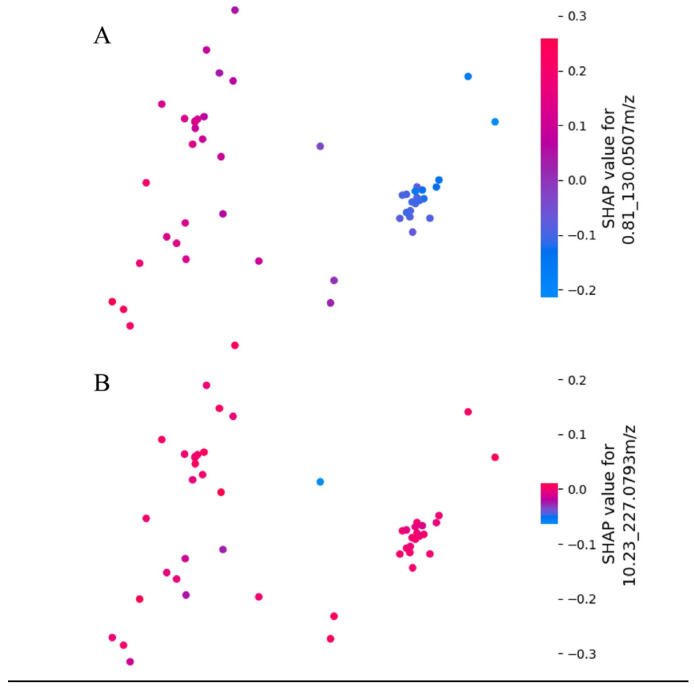
SHAP embedding plots. (**A**) Embedding plot highlighting 0.81_84.0447*m*/*z*. (**B**) Embedding plot highlighting 10.23_227.0793*m*/*z*.

Additionally, SHAP values were projected into a two-dimensional space using PCA, allowing for the visualization of the impact of metabolomic features. This approach helps observe both the intensity of SHAP values and the clustering of positive and negative SHAP values. Figure 7A displays the SHAP embedding plot for the feature “0.81_130.0507*m*/*z*,” highlighting the clustering of positive and negative SHAP values. In contrast, Figure 7B shows the embedding plot for “10.23_227.0793*m*/*z*,” identified as the least important feature.

**Figure 8:**
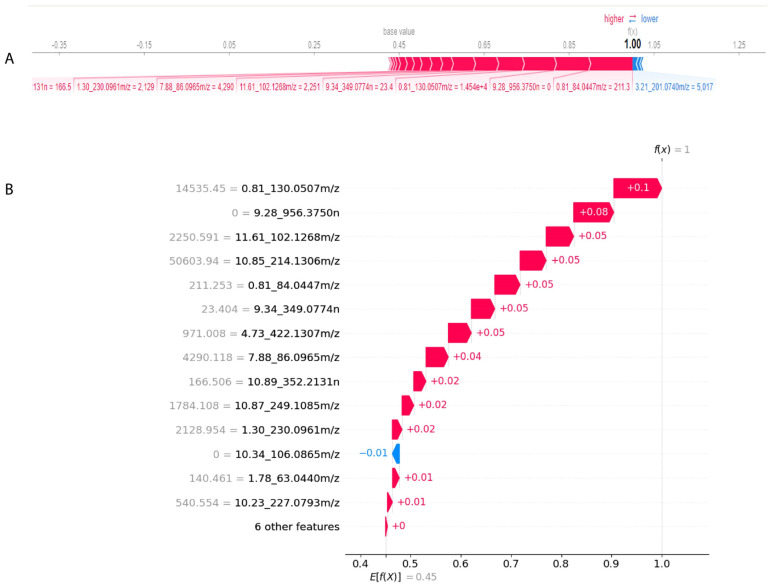
Local explanations of a representative sample. (**A**) Force plot showing an influenza prediction. (**B**) Waterfall plot displaying the same prediction.

SHAP offers various methods for generating local explanations (i.e., explanations for individual samples), including the waterfall plot and force plot visualizations. Figure 8A illustrates a force plot, while Figure 8B shows a waterfall plot. In the waterfall plot (Figure 8B), the *x*-axis denotes the probability of a sample being classified as male, and the *y*-axis displays the metabolomic features along with their respective values for the sample. The plot starts with the model’s expected value on the *x*-axis, which is E[f(X)] = 0.45. This ‘base’ value, 0.45, represents the average prediction probability across the test set. The plot then shows how the metabolomic features influence the model’s output. Positive contributions (shown in red) and negative contributions (shown in blue) adjust the expected value to the final model output, which is f(x) = 1. Positive SHAP values increase the likelihood of the sample being classified as influenza, while negative SHAP values decrease this probability. Likewise, the force plot visualizes the SHAP values of each feature for a specific sample using an additive force layout (Figure 8A).

Hierarchical clustering, shown in Supplementary Figure S3, has a horizontal axis for features and a vertical axis for samples. This method helps identify patient subgroups and predict outcomes. Each cell’s color shows the average relative abundance of a feature in a sample, with darker shades indicating higher abundance. Hierarchical clustering organizes features and samples based on similarity, grouping them at the top and bottom of the heatmap. This visual tool helps recognize patterns, revealing possible links between highly expressed features and specific biological processes in sample groups.

## 4. Discussion

Influenza, a widespread respiratory infection affecting the sinuses, lungs, and throat, remains a global health concern with an annual incidence of approximately 5–10% in adults and 20–30% in children. In the context of influenza, A viruses (IAVs), characterized by antigenic drift, early detection poses a challenge because of shared symptoms with other respiratory infections. Despite advancements in diagnostic methods, such as PCR-based tests, limitations persist, including issues of sensitivity, cost, and the inability to distinguish active infection from latency or colonization. Innovative strategies are essential to overcome these challenges and enhance timely intervention for effective anti-influenza treatments.

The application of machine learning in metabolomics is quite fascinating. ML-based algorithms have been integrated into the analysis of the nasopharyngeal metabolome in COVID-19, influenza, and RSV infections [55]. Similarly, Gerard et al. [30] employed metabolomics and ML to analyze metabolite concentrations in COVID-19 patients’ serum and urine, underscoring ML’s potential in disease diagnosis and management. An exploration of acute ischemic stroke underscored the crucial role of machine learning in predicting functional recovery outcomes by analyzing metabolomics data and clinical characteristics. This highlights the versatile application of machine learning in advancing medical research. This study employed an integrated machine learning (ML) approach to predict influenza using a publicly available dataset of nasopharyngeal metabolomics. The dataset comprised 236 nasopharyngeal swab samples, of which 118 were positive for the influenza viruses (2009 H1N1, H3N2, and Flu B) and 118 were negative. We utilized t-SNE and parallel coordinate plots to evaluate and visualize the quality of the reduced feature space. The t-SNE plots, shown in Figure 2(ci), effectively illustrate the discriminative power of the top 20 features selected using the SHAP (Shapley Additive Explanations) method. These plots demonstrate a clear separation between the influenza-positive and influenza-negative samples, indicating the efficacy of the selected features in distinguishing between the two classes. The parallel coordinate plot, shown in Figure 2d, further supports this by illustrating the high separability of the two classes. It displays the normalized abundance values of various compounds, revealing a wide range of mass-to-charge (*m*/*z*) values and intensities. This indicates a mixture of compounds with similar structures in the samples, with certain compounds showing distinct differences in intensity between the influenza-positive and negative samples. These visualizations confirm the robustness of the selected features and their potential for accurate classification of influenza status in prospective sample cohorts.

The stacking-based meta-models demonstrate superior efficacy compared with the individual models, as shown in Table 1. The top three models CatBoost, RandomForest, and AdaBoost were employed to create the stacking models using their probabilities. The best stacking meta-model was ExtraTrees, which achieved an accuracy of 97.08%, precision of 97.11%, recall of 97.08%, specificity of 97.18%, F1 score of 97.08%, and AUC of 98.12%. We also conducted external validation. The 96 samples included 14 from children, 80 from adults, and 3 with documented viral coinfections (seasonal coronavirus, RSV, or CMV). For this external dataset, the stacking model achieved a perfect score of 100% across all metrics. This stacking model outperformed the previous study by Hogan et al. [28], as detailed in Table 2.

The application of machine learning in metabolomics presents a powerful approach to the early detection and differentiation of influenza infections. By analyzing complex datasets and identifying subtle patterns, ML models can enhance diagnostic accuracy and support timely intervention and effective antiviral treatments. Model explainability played a pivotal role in this study. SHAP analysis identified key metabolites influencing the model’s predictions, such as 0.81_84.0447*m*/*z* (Pyroglutamic Acid) and 0.81_130.0507*m*/*z* (In-Source Fragment Ion of Pyroglutamic Acid), which exhibited lesser contributions in influenza-positive patients. Conversely, metabolites like 10.34_106.0865*m*/*z* and 8.65_211.1376*m*/*z* demonstrated significant positive contributions.

## 5. Limitations

The current study has several limitations. Firstly, this study relies on Hogan et al.‘s [28] nasopharyngeal Metabolomics dataset, comprising 236 nasopharyngeal swab samples. While the dataset is meticulously described, the limitation arises from its relatively small size. This size constraint is particularly notable during the SHAP analysis, which offers valuable insights into feature contributions to the model’s output. The analysis points out specific features’ roles in influenza positivity but emphasizes the need for caution because of the small dataset size. To ensure more robust and reliable analyses, a larger dataset is needed. As this study compares its results to Hogan et al.’s [28] work, it highlights the superior performance of the stacking-based models, achieving perfect sensitivity, specificity, and AUC. However, the effectiveness of the model, while promising, should be interpreted with consideration of the dataset’s limited size. Future directions for this research should prioritize the acquisition of a more extensive dataset to enhance the model’s generalizability and reinforce the credibility of the findings. While this dataset does not report patients with coinfections, it was not possible to investigate how the proposed model can distinguish the coinfections. It is crucial to acknowledge that ML models may face challenges in differentiating among infections with shared symptoms. Future research should aim to enhance the specificity of ML algorithms in identifying coinfections. Our study offers notable advantages, including faster turnaround time and higher accuracy compared with conventional methods. The diagnostic test, compatible with existing sample collection practices and laboratory infrastructure, utilizes nasopharyngeal swab samples and LC/Q-TOF mass spectrometry [56]. Our future work will focus on step-by-step integration into clinical practice, including training technicians, acquiring the necessary equipment, implementing the ML model within existing laboratory information systems, and evaluating scalability and cost-effectiveness. Additionally, we will pursue rigorous clinical trials and regulatory approval, conduct larger validation studies, and perform comprehensive cost-effectiveness analyses to ensure broad clinical adoption and robust performance. Additionally, our next objective will be to work on a large-scale influenza dataset. We plan to collect custom influenza metabolomics data. The dataset used in our study comprised 118 influenza-positive cases (36 H1N1, 39 FLU B, 43 H3N2). Limited data on coinfections posed a challenge for our ML model. Future research will focus on classifying patients with coinfections, as larger datasets are crucial for enhancing model performance.

Despite these limitations, this study significantly contributes to the exploration of ML applications in influenza prediction and provides a valuable foundation for future endeavors with larger datasets. Another limitation is the testing for other viruses. More than 20 pathogens might cause respiratory illness. A comparison between all of them is needed for full validation [57].

## 6. Conclusions

In our study, we introduced a stacking-based approach leveraging multiple machine learning models to enhance the precision of influenza prediction using metabolomics data. Our methodology was evaluated using a publicly available dataset of nasopharyngeal metabolomics, encompassing samples from individuals both positive and negative for influenza viruses. The results obtained from our primary dataset showcased promising outcomes. Our top-performing stacking meta-model, employing ExtraTrees, achieved notable metrics including accuracy, precision, recall, specificity, F1 score, and area under the curve (AUC), achieving an accuracy of 97.08%. Furthermore, during external validation using a diverse dataset comprising samples from children, adults, and individuals with viral coinfections, our stacking model demonstrated perfect performance across all metrics—100% sensitivity, specificity, and AUC. This performance surpasses previous benchmarks set by comparative studies. The interpretability of our model was pivotal for comprehending feature impacts on predictions. Through SHAP (Shapley Additive Explanations) analysis, we identified critical metabolites influencing the model’s decisions. Notably, metabolites such as 0.81_84.0447*m*/*z* (Pyroglutamic Acid) and 0.81_130.0507*m*/*z* (In-Source Fragment Ion of Pyroglutamic Acid) exhibited minimal contributions in influenza-positive patients, whereas others like 10.34_106.0865*m*/*z* and 8.65_211.1376*m*/*z* demonstrated significant positive contributions. In future research, evaluating the performance of methods in cases of coinfections—where individuals have multiple respiratory viruses—will be crucial. Distinguishing between similar symptoms of influenza and other infections requires a comprehensive dataset encompassing various respiratory pathogens. This will enable improvements in diagnostic accuracy, understanding of pathogen interactions, and clinical management for respiratory illnesses.

## Figures and Tables

**Table 1:** Results for the 13 ML models and stacked ML model.

	Individual ML Model						Stacked ML Model (With Different Meta-classifiers)					
Models	Acc	P	R	SP	F1	Auc	Acc	P	R	SP	F1	Auc
**CatBoost**	**94.58**	**94.58**	**94.58**	**94.59**	**94.58**	**98.57**	95.83	95.83	95.83	95.82	95.83	98.78
**RandomForest**	**94.16**	**94.16**	**94.16**	**94.14**	**94.16**	**97.74**	96.25	96.25	96.25	96.27	96.25	98.21
**AdaBoost**	**93.75**	**94.01**	**93.75**	**94.04**	**93.75**	**98.34**	95.42	95.45	95.42	95.5	95.42	97.19
**ExtraTrees**	93.75	93.75	93.75	93.69	93.74	97.52	**97.08**	**97.11**	**97.08**	**97.18**	**97.08**	**98.12**
**LogisticRegression**	92.91	93	92.91	93.06	92.91	93.27	95	95	95	94.98	95	98.3
**ElasticNet**	92.91	93	92.91	93.06	92.91	94.73	95	95	95	94.98	95	98.3
**SVM**	92.5	92.5	92.5	92.47	92.5	93.54	95.83	95.83	95.83	95.82	95.83	98.28
**LDA**	91.25	91.24	91.25	91.18	91.24	96.23	95.42	95.45	95.42	95.5	95.42	97.99
**LGBM**	91.25	91.26	91.25	91.11	91.24	95.87	95.42	95.42	95.42	95.43	95.42	98.27
**MLPClassifier**	90.83	91.04	90.83	91.07	90.83	94.85	95	95	95	94.98	95	97.48
**XGBClassifier**	90.83	90.83	90.83	90.73	90.82	94.34	94.58	94.62	94.58	94.67	94.59	97.92
**Kneighbors**	89.58	89.61	89.58	89.65	89.58	94.55	94.58	94.59	94.58	94.6	94.58	96.45
**GradientBoost**	89.58	90.097	89.58	89.99	89.57	95.04	94.17	94.17	94.16	94.08	94.16	98.65

Bold values represent the best results among all the models.

**Table 2:** Comparison of evaluation metrics with other work (external validation).

	Model	Sensitivity	Specificity	AUC
Hogan et al. [28]	LGBM	94%	96%	100%
**Ours**	**Stacking-based Extra Trees**	**100%**	**100%**	**100%**

## Data Availability

The dataset used in this study can be accessed from the following link: https://github.com/stanfordmlgroup/influenza-qtof/tree/master/data. (accessed on 12 December 2023).

## Supplementary Materials

The following supporting information can be downloaded at https://www.mdpi.com/article/10.3390/diagnostics14192214/s1: Supplementary Figure S1: ROC Curve for Best Stacking Model (ExtraTrees) in 5 Folds. Supplementary Figure S2: Confusion Matrix of the best stacking model (Etratrees). Supplementary Figure S3: Hierarchically Clustered Heatmap of Average Relative Abundance Values for Selected Features (Negative = 0, H1N1 = 1, FluB = 2, H3N2 = 3). Supplementary Table S1: Demographic characteristics of all patients.

## Abbreviations

APACHE II	Acute Physiology and Chronic Health Evaluation II
AUC	area under the curve
COVID-19	coronavirus disease 2019
GC/MS	gas chromatography–mass spectrometry
HCC	hepatocellular carcinoma
IAVs	Influenza A viruses
IOKR	Input–Output Kernel Regression
LC/MS	liquid chromatography–mass spectrometry
LC/Q-TOF	liquid chromatography–quadrupole time-of-flight mass spectrometry
LGBM	light gradient-boosting machine
ML	machine learning
PCA	Principal Component Analysis
PCR	polymerase chain reaction
RF	Random Forest
RSV	respiratory syncytial virus
SHAP	Shapley Additive Explanations
SWQS	weighted quantile sketch
T-SNE	t-distributed Stochastic Neighbor Embedding
WHO	World Health Organization
