# Supplementary material for: Enhancing Influenza Detection through Integrative Machine Learning and Nasopharyngeal Metabolomic Profiling: A Comprehensive Study
Source: Diagnostics (Basel). 2024 Oct 4;14(19):2214. doi: 10.3390/diagnostics14192214 (PMC11476346; doi:10.3390/diagnostics14192214)
Supplement: Supplementary file 1 [file diagnostics-14-02214-s001.zip › diagnostics-3201210-supplementary.pdf]

Supplementary Materials

Supplementary Table S1 : Demographic characteristics of all patients.

		**Non-influenza (n = 118)**	**Influenza (n = 118)**	**p-value***
Age (No. [%])	>=2yo-17yo	48 (40.7%)	48 (40.7%)	1
	>=18yo	70 (59.3%	70 (59.3%)	
Sex (No. [%])	Male	62 (52.5)	61 (51.7)	0.9
	Female	56 (47.5)	57 (48.3)	
Immunocompromised (No. [%]	Yes	54 (45.8%)	27 (22.9%)	0.001
	No	63 (53.4%)	87 (73.7%)	
	Unknown	1 (0.8%)	4 (3.4%)	
Comorbidities (No. [%])	Leukemia/lymphoma	27 (22.9%)	10 (8.5%)	0.005
	Active malignancy	10 (8.5%)	2 (1.7%)	0.02
	Asthma	6 (5.1%)	7 (5.9%)	0.5
Median Charlson comorbidity index score (IQR)	1 (0-3)	0 (0-2)	0.002	
Days of symptoms at the time of testing (mean; SD)	3 (1-7)	3 (2-9)	0.4	
Patient location (No. [%])	ED	41 (34.8%)	36 (30.5%)	<0.001
	ICU	16 (13.6%)	4 (3.4%)	
	Inpatient ward	30 (25.4%)	3 (2.5%)	
	Outpatient clinic	31 (26.3%)	75 (63.6%)	
Antiviral treatment at time of testing (No. [%])	Yes	0	3 (2.5%)	0.1
	No	114 (96.6%)	96 (81.4%)	
	Unknown	4 (3.4%)	19 (16.1%)	
Antibiotic treatment at time of testing (No. [%])	Yes	16 (13.6)	38 (32.2)	<0.001
	No	82 (69.5%)	76 (64.4)	
	Unknown	20 (17.0)	4 (3.4)	
Confirmed bacterial coinfection or colonization at time of testing (No. [%])	Yes	8 (6.7)	38 (32.2)	<0.001
	No	110 (92.4	76 (64.4)	
	Unknown	1 (0.8)	4 (3.4)	
Hospitalization (No. [%])	Yes	82 (69.5%)	29 (24.6%)	<0.001
	No	36 (30.5%)	89 (75.4%)	
ICU admission (No. [%])	Yes	26 (22.0%)	6 (5.1%)	<0.001
	No	92 (78.0%)	112 (94.9%)	
30-day all-cause mortality (No. [%])	Yes	3 (2.5%)	3 (2.5%)	1
	No	115 (97.5%)	116 (97.5%)	

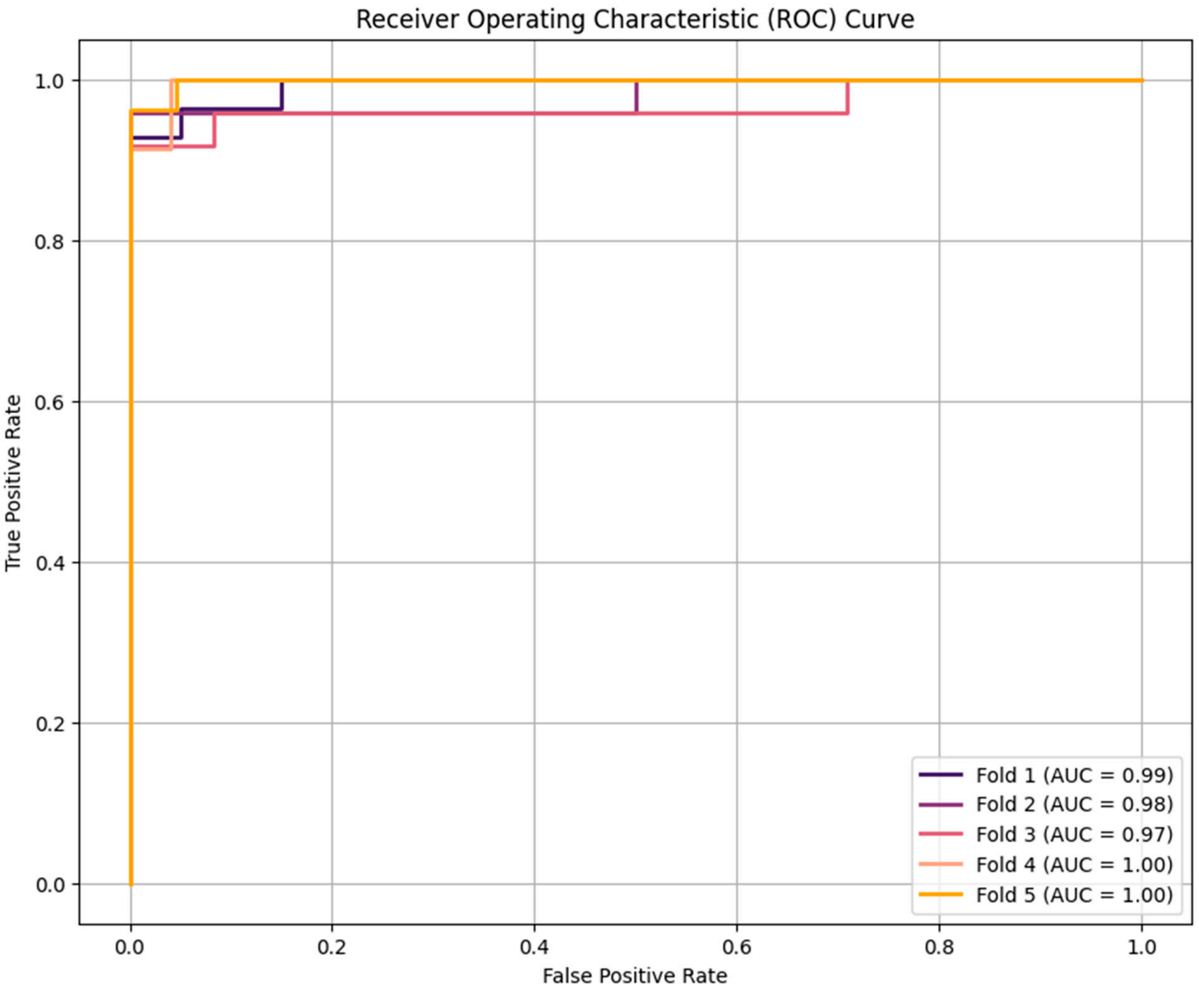

Supplementary Figure S1: ROC Curve for Best Stacking Model (ExtraTrees) in 5 Folds.

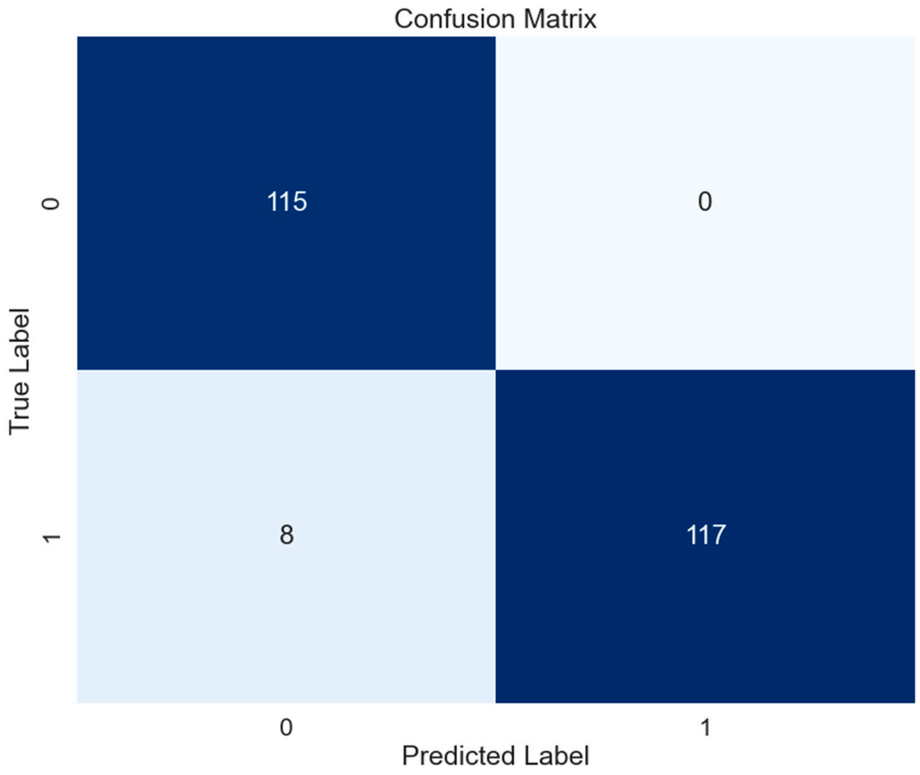

Supplementary Figure S2: Confusion Matrix of the best stacking model (Etratrees).

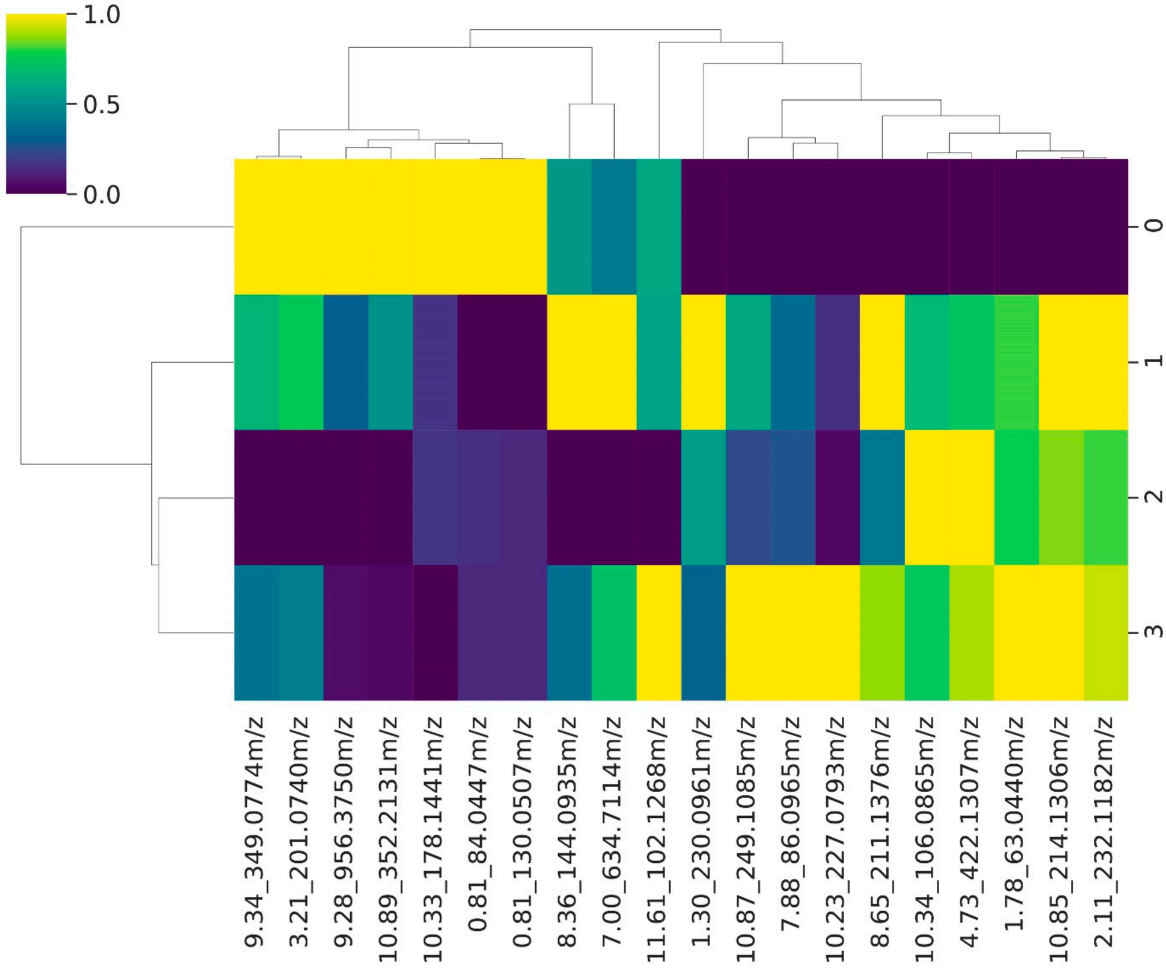

A

Supplementary Figure S3: Hierarchically Clustered Heatmap of Average Relative Abundance Values for Selected Features (Negative=0, H1N1=1, FluB=2, H3N2=3)
